# Exclusion Performance in Dwarf Goats (*Capra aegagrus hircus*) and Sheep (*Ovis orientalis aries*)

**DOI:** 10.1371/journal.pone.0093534

**Published:** 2014-04-02

**Authors:** Christian Nawroth, Eberhard von Borell, Jan Langbein

**Affiliations:** 1 Department of Animal Husbandry and Ecology, Institute of Agricultural and Nutritional Sciences, Martin-Luther-University, Halle, Germany; 2 Institute of Behavioural Physiology, Leibniz Institute for Farm Animal Biology, Dummerstorf, Germany; Queen Mary, University of London, United Kingdom

## Abstract

Using a comparative approach, we investigated the ability of dwarf goats and sheep to use direct and indirect information about the location of a food reward in an object-choice task. Subjects had to choose between two cups with only one covering a reward. Before making a choice, subjects received information about the baited (direct information) or non-baited cup (indirect information). Both goats and sheep were able to use direct information (presence of food) in the object choice task. After controlling for local enhancement, we found that goats rather than sheep were able to use indirect information (i.e., the absence of food) to find a reward. The actual test setup could not clarify whether individual goats were able to inferentially reason about the content of the baited cup when only shown the content of the non-baited cup or if they simply avoided the empty cup in that situation. As browsing species, feral and wild goats exhibit highly selective feeding behaviour compared to the rather unselective grazing sheep. The potential influence of this species-specific foraging flexibility of goats and sheep for using direct and indirect information to find a food reward is discussed in relation to a higher aversion to losses in food acquisition in goats compared to sheep.

## Introduction

A fundamental question in comparative studies of the cognitive abilities of non-human animals is to distinguish between a gradual development in performance due to associative learning mechanisms and complex cognition, such as sudden insightful solutions [1]. Individuals exhibit “insight” to solve a new problem when mental reorganisation of the problem leads into a sudden solution without trial-and-error learning [2]. Although in normal life it is difficult to unambiguously rule out associative explanations for insightful behaviour (e.g., [3]), different experimental setups have been used to provide evidence of behavioural reactions that rely on processes that are more consistent with reasoning rather than learning [4].

Inferential reasoning, in particular, implies the establishment of an association between a visible and an imagined event [1]. The subject selects the correct solution by excluding other potential alternatives even though only indirect information is available. However, inferential reasoning can only be assumed if the subject exhibits adequate behaviour right from the start, without explicit training. Otherwise it's hard to rule out associative learning [5], [6]. Different experimental setups have been used to study inferential reasoning by exclusion in animals. One example is to train animals on a set of items where each is associated with a specific label. By introducing a new item and giving the animal the choice between a familiar and a new label, subjects infer by choosing the new label that it must refer to the new item [7], [8]. Another approach frequently used to study inferential reasoning is the matching-to-sample paradigm, in which a subject is trained to a conditional discrimination [9]. When a new undefined sample is introduced, the subject must choose between a novel and a familiar comparison. The matching to sample procedure has been applied to test inference by exclusion in chimpanzees, sea lions, bottlenose dolphins and pigeons [10]–[14]. Some of these experimental approaches have been criticised because the artificial setting hampers animals from exhibiting spontaneous behaviour. Furthermore, it requires massive pre-training of the animals, and it is often difficult to exclude the possibility that they simply acted on the basis of previously learned associations [6], [15], [16].

In reflection of these critiques, Premack and Premack [17] designed a simple food-finding task to study exclusion behaviour that is more naturalistic and requires no pre-training of the subjects. They presented primates with two boxes in which, visible to the test subject, two different types of a reward (banana or apple) were hidden. Later, the subject witnessed the experimenter eating one of the rewards. The question was whether the subject could infer from this information which box still contained the reward. Call [18] has slightly modified this protocol for the use with different primate species, in which the subjects were presented with two opaque cups of which only one was baited. Then, the subjects were given information about the content of both cups (full information), about the baited cup (direct information), about the non-baited cup (indirect information) or no information at all. In the case of providing indirect information, it can be tested whether the subjects are able to choose the location of the hidden reward. It was argued that this visual version of the cup task cannot distinguish between the underlying processes needed to solve the task [18] because subjects could either use inferential reasoning (high-level explanation) or a simple avoidance of the empty cup (low-level explanation). According to previous work [16], [19], [20], we therefore refer to the performance in the cup task with the term ‘exclusion performance’ to cover both the low- and high-level, explanations.

Throughout the last decade, the experimental design applied by Call [18] and variants of this two-way object-choice task have been used with primates, dogs and birds to study exclusion performance, allowing direct interspecies comparisons [4], [15], [16], [18], [19], [21]–[30]. Current research in animal cognition suggests that either all species share general mechanisms of learning and problem solving due to their common phylogenetic history [31] or every species possesses a specific set of cognitive abilities, adaptive to their specific ecological and social environments [32], [33]. Differences in decision-making between two or more closely related species can often be linked to their specific feeding ecology. For instance, comparative studies of apes showed that chimpanzees (*Pan troglodytes)* are more risk-prone than bonobos (*Pan paniscus)* in a choice task where they had to choose between a safe, but lower-valued, and a risky, but higher-valued, reward [34], [35]. The use of extractive skills to capture prey might explain, at least in a modified cup task using auditory cues instead of visual information, why great apes and capuchin monkeys (*Cebus paella)* solve the acoustic version of the cup task whereas other primate species do not [15], [18], [23], [24], [28].

In a comparative approach, caching in corvids was linked to their ability to solve a visual exclusion task [16], [19], [20]. Caching species such as Raven (*Corvus corax)* [16] and Carrion Crows (*Corvus corone corone)* [20] were successful in choosing the baited cup when only indirect information was provided, whereas the Jackdaw (*Corvus monedula)*, a corvid species that caches only occasionally, was not capable of solving the task using direct or indirect methods [19]. However, a recent study of the Eurasian Scrub Jay (*Garrulus glandarius)*, a highly specialised cacher, showed no positive results, challenging the relationship between caching and exclusion performance [27]. Another potential explanation for the performances of different species may be linked to a more general aspect of a specieś feeding ecology. For instance, a difference in foraging flexibility or a differing sensitivity to losses in food acquisition may account for different performances as well. However, no studies have explicitly focussed on these differences in former comparative studies using the cup task.

Investigating the cognitive abilities of small ruminants (e.g., goats and sheep) is of interest on several levels. First, from a comparative point of view, a close phylogenetic relationship of two species with characteristic differences in their feeding ecology can shed light on the evolutionary forces that shape certain cognitive skills (see above). Second, domesticated ruminants live in artificial environments and therefore must cope with different challenges than non-domesticated ruminants living in the wild. From an applied view, it is therefore necessary to understand how domestic animals perceive and respond to their physical world to adjust these artificial environments according to their needs.

In this study, we investigated the performance of dwarf goats and sheep in a visual exclusion task that has previously been conducted with primates, birds, and dogs. Although most cognitive research on small ruminants has investigated their discriminatory learning abilities (goats: [36]–[38], sheep: [39], [40]) some studies have investigated other cognitive aspects, such as gaze following [41], parent-offspring recognition [42] and cognitive bias [43]–[45]. Goats and sheep are closely related species, but differ in their foraging behaviour [34], [35]. Whereas goats are dietary browsers and prefer low-fiber plant material, such as stems and leaves, sheep as dietary grazers rely primarily on high-fiber plants, such as grass [46]. That means that although goats are able to digest grass, which contains a higher level of cellulose, they are more selective in their feeding behaviour than sheep. We predicted that goats, being more flexible in their food acquisition, outperform sheep in avoiding the empty food container and choosing the baited one.

## Materials and Methods

### Ethics statement

All procedures involving animal handling and treatment were approved by the Committee for Animal Use and Care of the Ministry of Agriculture, the Environment and Consumer Protection of the federal state of Mecklenburg-Vorpommern, Germany (Ref. Nr. 7221.3-2.1-008/12). Housing facilities met the German welfare requirements for farm animals.

### Subjects and housing

Twelve Nigerian dwarf goats (aged from 3–4.5 years; all female) and six East Friesian dairy sheep (approximately 2 years of age; all female) participated in two consecutive experiments. Goats were group-housed at the Leibniz Institute for Farm Animal Biology. Sheep were group-housed at a private farm close to Leipzig, Germany. All animals were housed indoors on straw bedding, received food and water ad libitum and were not food-restricted in any phase of the experiments. Throughout the testing period, all rooms were lit by natural light, supplemented by artificial light during test sessions. The goats participated in a study of visual discrimination learning using a fully automated learning device at the age of six months [38]. The sheep had no previous test experience. None of the animals had participated in an object choice task before this study was conducted. Sheep were tested at noon on a daily basis in January 2012. Goats were tested from 9:00–12:00 and, optionally, from 14:00–17:00 on a daily basis in April 2013.

### Materials

**Figure 1:**
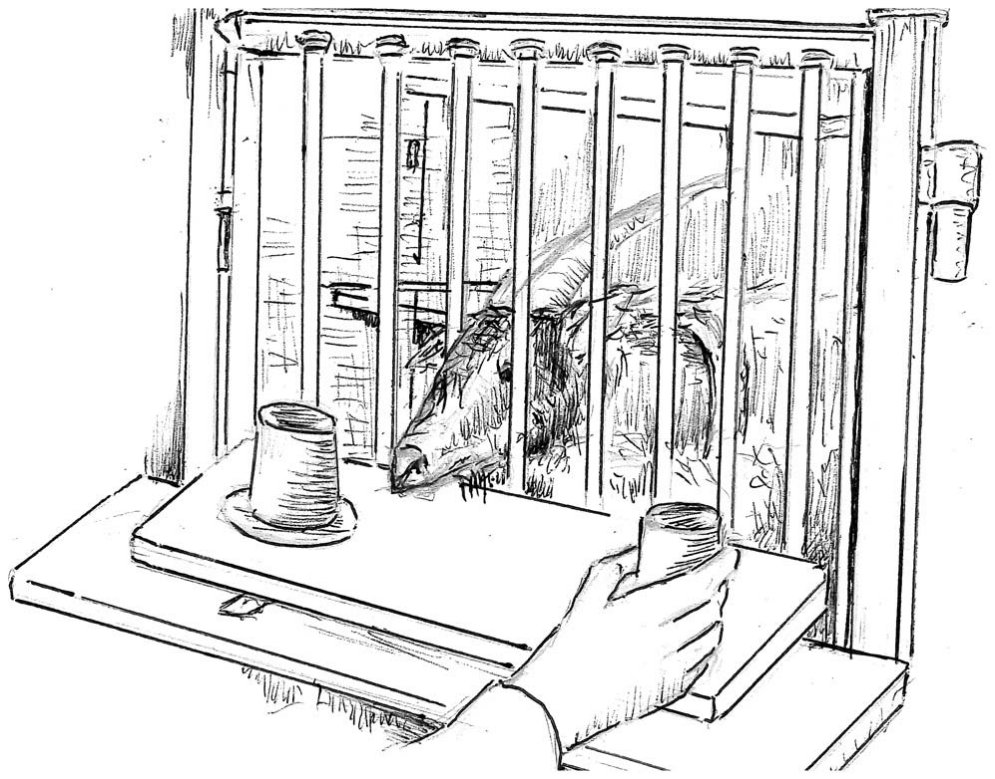
Illustration of the testing apparatus and subject position during testing.

For training and testing, the goats were separated in a compartment adjacent next to their home pen (150 cm × 125 cm). The sheep were separated from the group in a single pen (120 cm × 270 cm). All test subjects were visually isolated from their pen mates, but had auditory and olfactory contact with their companions at all times. The experimenter was seated in another compartment, separated from the test animal by a mesh, leaving the subjects several spaces within the mesh where they could indicate a choice (Fig. 1). A sliding table (60 cm × 25 cm) was placed in front of the mesh. For the dwarf goats, it was placed on the ground, and for the sheep, it was placed on a small table with a height of approximately 35 cm. In training and testing, two dark brown bowls (diameter: 14 cm) were placed on the board with a distance of 35 cm. Two dark brown cups (diameter: 11 cm; height: 10 cm) were used to cover the bowls. The distance between the bowls and the subject was approximately 30 cm.

### Procedure

#### Shaping

Shaping was introduced to habituate the subjects to the test procedure and to train them how to indicate a choice. In shaping trials, one flat plastic bowl was located in the middle of the sliding board. In the first four trials of a shaping session, the experimenter (E) put a food reward (for goats: a piece of uncooked pasta; for sheep: a slice of sugar beet) into the bowl and then pushed the platform towards the mesh to let the subject make its choice. If the animal put its nose through one of the middle gaps in the mesh, it obtained a reward. This was repeated for eight additional trials, but for these, the E covered the bowl with a cup before letting the subject make its choice. Shaping sessions were repeated until the individual showed no signs of arousal or stress during participation and instantly chose the baited position. Subjects who did not meet these criteria after the third shaping session were excluded (one goat). Two goats required two shaping sessions, and one goat required three sessions. All other individuals met the criteria after the first session. Therefore, six sheep and eleven goats proceeded to the training.

#### Training

Training sessions were conducted once or twice a day and consisted of ten trials each. Two bowls were placed on the left and right sides of the sliding board at a distance of 35 cm. The E baited only one bowl in full view of the subject, covered both bowls with cups after baiting, and pushed the board towards the mesh. Each side was baited pseudorandomly five times per session. The subject made its choice by putting its snout through one of the outer left or right gaps in the mesh and obtained the reward only if it chose correctly. Subjects were considered to have completed training when they achieved at least eight out of ten correct choices in two consecutive sessions (binomial test; P = .012). One goat required four and one sheep three training sessions. All other individuals met the criterion after the second session and proceeded therefore to the test.

### Experiment 1 - choice by exclusion

Before each test session, subjects received two further training trials to ensure motivation. The procedure in the test trials was similar to that in the training trials except that the subject never saw the baiting procedure, which was conducted outside of the subjects view. After the E placed both bowls, each covered by a cup, on the sliding board, the subject received one of four different test conditions with different information provided:

**Conditions.**

1. both – E lifted both cups simultaneously for approximately 5 seconds, giving full information to the subject;
2. baited - E lifted the baited cup for approximately 5 seconds while simultaneously touching the non-baited cup, giving only direct information to the subject;
3. empty – E lifted the non baited cup for approximately 5 seconds while simultaneously touching the baited cup, giving only indirect information to the subject;
4. control – E touched both cups simultaneously without lifting them for approximately 5 seconds, giving no information to the subject.

Subjects received ten test sessions of eight trials each (two trials for every condition in each session) with a total of 20 trials of each condition. The left and right bowls were baited pseudorandomly, with the restriction that no side was baited more than two times consecutively. Depending on the subjectś motivation, they received either one or two sessions in a row.

### Experiment 2 - control for local enhancement

In experiment 1, subjects could simply be distracted due to local enhancement effects and could therefore be biased to choose the cup that was lifted by the experimenter. To exclude this possibility, some researchers [19]–[21] have introduced a slightly modified setup where in every condition two additional inner cups (either transparent or opaque) are used and two outer cups are lifted simultaneously. Thus, using different combinations of opaque or transparent inner cups, the information level of the conditions of experiment 1 can be replicated while excluding local enhancement effects. Here, the procedure was the same as in experiment 1 except that underneath the outer cups two smaller cups, either transparent or opaque, were located. The conditions were the same as in the first experiment except that in every condition both outer cups were lifted to avoid local enhancement effects. Reproducing the four informational levels described in experiment 1, the inner cups were either opaque or transparent.

**Conditions.**

1. both – E lifted both outer cups simultaneously for approximately 5 seconds; both inner cups were transparent (full information)
2. baited - E lifted both outer cups simultaneously for approximately 5 seconds; the inner baited cup was transparent, the inner non-baited cup was opaque (direct information)
3. empty – E lifted both outer cups simultaneously for approximately 5 seconds; the inner baited cup was opaque, the inner non-baited cup was transparent (indirect information)
4. control – E lifted both outer cups simultaneously for approximately 5 seconds; both inner cups were opaque (no information)

All other circumstances were the same as in experiment 1.

### Data analysis

All trials were coded live and were videotaped (goats: Panasonic WV-CP500 and HDCCTV Digital Video Recorder EDRHD-4H4; sheep: Camcorder JVC F1.2). A trial was scored as ‘correct’ if the subject chose the baited cup (see supplementary material, videos S1, S2, and S3). All choices could be classified unambiguously as correct or incorrect, so we did not calculate inter-observer reliability. Performance in the choice test was modelled using a generalised linear mixed model. Therefore, we used PROC GLIMMIX (SAS 9.2, SAS Institute Inc., Cary, NC, USA) for a binary distribution and logit as link function to analyse the impact of ‘species’ (2 levels), ‘condition’ (4 levels), ‘experiment’ (2 levels) and their corresponding two-way interactions. Two factors, ‘condition’ and ‘experiment’, were modelled as repeated factors. Least square means (LSM) and their standard errors (SE) were calculated. For multiple comparison procedures (MCPs), adjustments for repeated testing were applied (Tukey–Kramer correction). For individual data on performance, binomial tests were conducted. If a subject chose the correct cup in at least 15 or more out of 20 trials for a given condition, the result was counted as significant (*P* = 0.041, two-tailed). To analyse potential learning effects at the group level, we compared the first against the last ten trials in every condition, using paired t-tests or exact Wilcoxon signed rank tests, as appropriate. The α - level was set at 5%.

## Results

**Figure 2:**
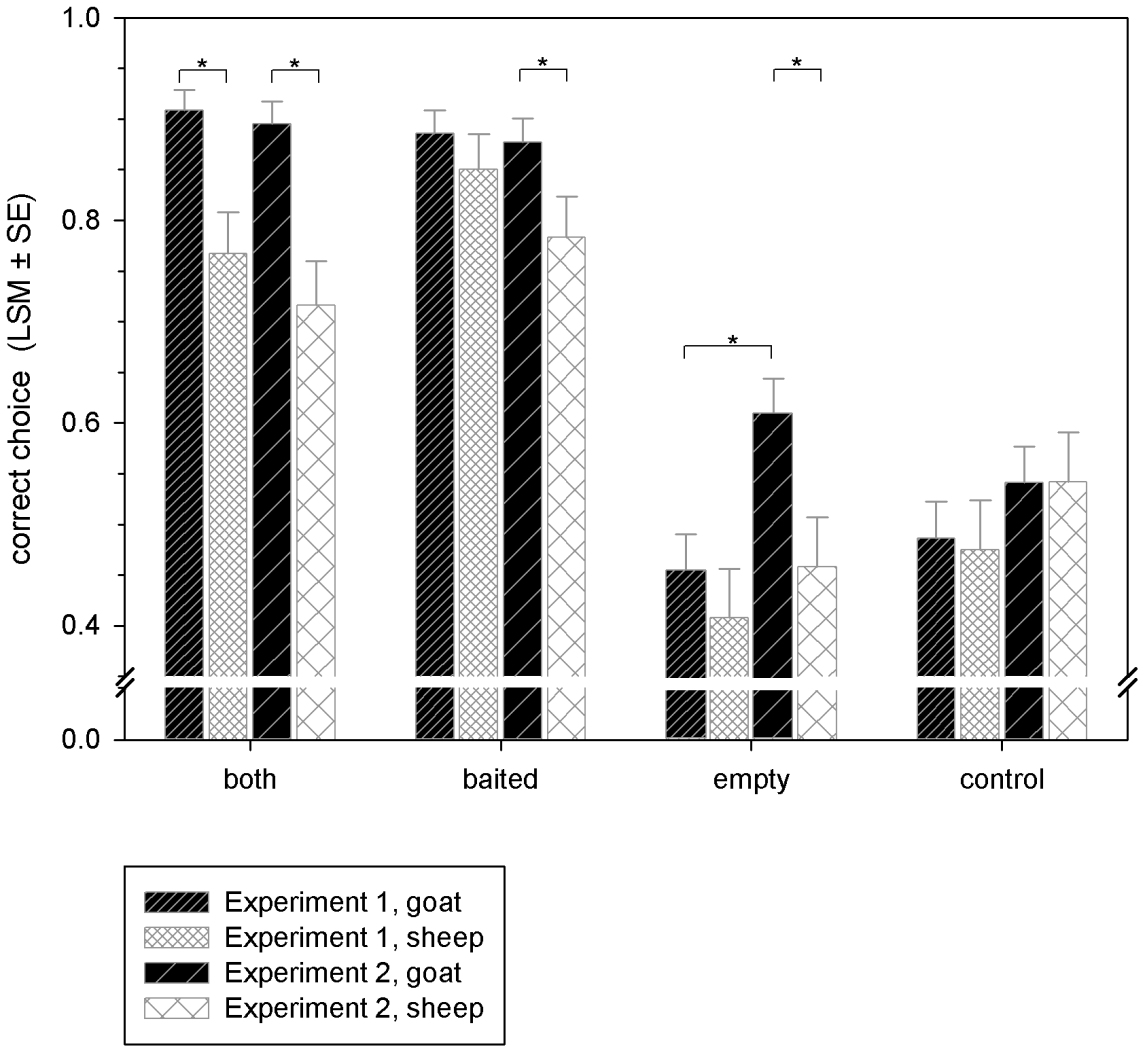
Least square means (± SE) of correct choices in the different test conditions in experiments 1 and 2 for goats and sheep. Subjects had to choose between two cups whereas only one was baited. Individuals were provided with full (‘both’), direct (‘baited’), indirect (‘empty’) or no information (‘control’) about the content of the two hiding locations. The corresponding cup(s) was/were lifted in experiment 1, whereas two inner cups (transparent or opaque) served as control for local enhancement effects in experiment 2 while both outer cups were lifted simultaneously in all test condition. Asterisks indicate significant differences between species and tests (P<0.05).

Of main interest was to analyse if the information provided across conditions (full, direct, indirect or no information), species and/or effects of local enhancement (experiment 1 vs. experiment 2) had an impact in solving the task. A three-way ANOVA indicated significant differences in the performances of goats and sheep (‘species’: F_1,120_ = 24.86, P<0.001) and an impact of the kind of information subjectś received (‘condition’: F_3,120_ = 89.61, P<0.001) as well as of the interaction between both factors on performance (‘species’ * ‘condition’: F_3,120_ = 5.31, P = 0.002). Additionally, a trend towards an interaction between experiments and the kind of information subjectś received occurred (‘experiment’ * ‘condition‘: F_3,120_ = 2.61, P = 0.055). Data from both experiments are illustrated in Figure 2.

### Experiment 1 – choice by exclusion

#### Goats

As a group, goats chose the rewarded cup significantly more often when both cups (‘both’) or only the baited cup (‘baited’) were lifted compared to the conditions were only the empty cup was lifted or no cup was manipulated at all (‘empty’ and ‘control’; MCP; all P<0.001). No other differences were found. On an individual level, all goats performed better than expected by chance when provided with full or direct information (binomial test; ‘both’ and ‘baited’: all P<0.05), whereas none exceeded chance level when provided with indirect or no information at all (‘empty’ and ‘control’: all P>0.05; see Table 1). When comparing the first 10 against the last 10 trials, goats only improved their performance when they received full information about the content of the cups (‘both’: mean ± SEM: first 10 trials: 8.55±0.37; last 10 trials: 9.64±0.20; exact Wilcoxon signed rank test; Z = −2.209; P = 0.039). No effect of learning was found for any other condition (paired t-tests or exact Wilcoxon signed rank tests; P>0.05).

**Table 1:** Number of correct trials (out of 20) for each individual across conditions in both experiments.

			Experiment 1				Experiment 2			
Subject	Species	age	both	baited	empty	control	both	baited	empty	control
**G1**	sheep	2 years	**18**	**17**	8	10	**15**	**18**	8	11
**G2**	sheep	2 years	**15**	**15**	12	9	**15**	**16**	10	11
**G3**	sheep	2 years	13	**17**	5	11	**15**	12	9	11
**R1**	sheep	2 years	12	**17**	7	7	13	**16**	10	11
**R2**	sheep	2 years	**20**	**18**	11	10	**17**	**20**	9	10
**R3**	sheep	2 years	14	**18**	6	10	11	12	9	11
**Mean**			15.33	17.00	8.17	9.50	14.33	15.67	9.17	10.83
**SEM**			1.26	0.45	1.14	0.56	0.84	1.31	0.31	0.17
**2**	goat	4 years	**19**	**19**	10	9	**18**	**18**	9	11
**3**	goat	3 years	**20**	**19**	12	11	**19**	**16**	14	12
**4**	goat	4 years	**20**	**18**	6	6	**17**	**19**	13	10
**5**	goat	4 years	**18**	**16**	12	8	**19**	**18**	11	11
**6**	goat	3 years	**17**	**17**	5	10	13	**15**	10	13
**7**	goat	3 years	**20**	**17**	4	11	**20**	**19**	7	10
**8**	goat	4 years	**17**	**17**	12	10	**17**	**17**	**17**	12
**9**	goat	4 years	**16**	**17**	7	11	**18**	**18**	13	9
**33**	goat	3 years	**18**	**18**	12	12	**20**	**19**	13	9
**44**	goat	3 years	**18**	**19**	10	11	**19**	**16**	**16**	11
**55**	goat	3 years	**17**	**18**	10	8	**17**	**18**	11	11
**Mean**			18.18	17.73	9.09	9.73	17.91	17.55	12.18	10.82
**SEM**			0.42	0.30	0.92	0.54	0.59	0.41	0.89	0.38

Experiment 1: standard two-way choice task where subjects were provided with full (both cups lifted), direct (baited cup lifted), indirect (empty cup lifted) or no information (control) about the content of two possible hiding locations; Experiment 2: control for local enhancement, accomplished by two additional inner cups. Significant performances are marked in bold (15 or more correct choices out of 20 trials; *P* = 0.041; binomial test, two-tailed).

#### Sheep

As a group, sheep chose the rewarded cup significantly more often when both cups or only the baited cup were lifted compared to the conditions were only the empty cup was lifted or no cup was manipulated at all (MCP; all P<0.001). No other differences were found. On an individual level, all sheep performed better than would be expected by chance when provided with direct information (binomial test; ‘baited’: all P<0.05), and three out of six sheep did so when provided with full information (binomial test; ‘both’: subject G1, P<0.001; subject G2, P = 0.041; subject R2, P<0.001; all other subjects: P>0.05). No individual exceeded the chance level when provided with indirect or no information at all (binomial test; ‘empty’ and ‘control’: all P>0.05; see Table 1). No effects of learning were found for any condition when comparing the first against the last ten trials (paired t-tests or exact Wilcoxon signed rank tests; all P>0.05).

#### Comparison between goats and sheep

The goatś performance exceeded that of the sheep when both received full information about the content of the cups (MCP; ‘both’: P = 0.002, see Fig. 2). No other differences were found.

### Experiment 2 – control for local enhancement

#### Goats

As a group, goats chose the rewarded cup significantly more often when both cups or only the baited cup were lifted compared to the conditions were only the empty cup was lifted or no cup was manipulated at all (MCP; all P<0.001). No difference between the two latter conditions was found (MCP; ‘empty’ vs. ‘control’: P>0.05). On an individual level, ten out of eleven goats performed above chance when provided with full information, and all subjects exceeded chance level when the received direct information (binomial test; all P<0.05; see Table 1). Contrary to experiment 1, two out of eleven goats performed above chance level when provided with indirect information only (binomial test; ‘empty’: subject 8, P = 0.003; subject 44, P = 0.012; all other subjects: P>0.05), whereas none exceeded chance level in the ‘control’ condition (binomial test; all P>0.05; see Table 1). No effects of learning were found for any condition when comparing the first against the last ten trials (paired t-tests or exact Wilcoxon signed rank tests; all P>0.05).

#### Sheep

As a group, sheep chose the rewarded cup significantly more often when both cups or only the baited cup were lifted compared to the conditions were only the empty cup was lifted or no cup was manipulated at all (MCP; ‘both’ vs. ‘empty’: P<0.001; ‘both’ vs. ‘control’: P = 0.011; ‘baited’ vs. ‘empty’: P<0.001; ‘baited’ vs. ‘control’: P<0.001). On an individual level, four out of six sheep performed above the level of chance when provided with full information (binomial test; ‘both’: subject G1, P = 0.041; subject G2, P = 0.041; subject G3, P = 0.041; subject R2, P = 0.003; all other subjects: P>0.05) and direct information (binomial test; ‘baited’: subject G1, P<0.001; subject G2, P = 0.012; subject R1, P = 0.012; subject R2, P<0.001; all other subjects: P>0.05). None of the sheep exceeded the chance level when provided with indirect or no information at all (binomial test; ‘empty’ and ‘control’: all P>0.05; see Table 1). No effects of learning were found for any condition when comparing the first against the last ten trials (paired t-tests or exact Wilcoxon signed rank tests; all P>0.05).

#### Comparison between goats and sheep

The goatś performance exceeded that of sheep when both received full, direct and indirect information about the content of the cups (MCP; ‘both’: P<0.001; ‘baited’: P = 0.039; ‘empty’: P = 0.015, see Fig. 2). No difference in performance was found when no information was provided at all (MCP; ‘control’: P>0.05).

### Comparison between performance in experiment 1 and 2

Due to the tendency towards an interaction between both experiments (‘experiment’) and the kind of information subjectś received (‘condition’), we also compared the performances of both species in both experiments. Across experiments, goats improved significantly in choosing the correct cup when they received indirect information about the content of both cups (MCP; ‘empty’: P = 0.003, see Fig. 2). No other differences were found (all P>0.05).

## Discussion

In this study, we applied a test setup first developed for primates [18] to investigate exclusion performance in dwarf goats and sheep. We investigated whether goats and sheep are able to use direct (presence of food) as well as indirect information (absence of food) to choose the correct location of a reward in an object-choice task. In both experiments, goats and sheep performed above the level expected by chance in the ‘both’ and ‘baited’ condition compared to a control, indicating that both species are able to use direct information in their choice behaviour. Therefore, the test paradigm in general appears to be suitable for testing small ruminants as well as corvids, dogs and primates [16], [18], [21]. At an individual level, nearly all goats and several of the sheep mastered the ‘both’ and ‘baited’ conditions in both experiments. Interestingly, goats and sheep were not able to use indirect information (i.e., the ‘empty’ condition) at either the group or the individual level in experiment 1. Similar negative results have been found for some corvid species and dogs when both cups were manipulated differently [19]–[21]. These negative findings were explained by local enhancement effects, i.e., the tendency to choose the cup that was manipulated last [20]. Thus, we changed the test design in experiment 2 to control for local enhancement effects. Here, goats outperformed sheep in the ‘both’, ‘baited’ and ‘empty’ condition. The individual data for the ‘empty’ condition point in the same direction. Two goats, but no sheep, chose the baited cup in this condition significantly above the chance level.

Although we cannot exclude the possibility that an increase in sample size would lead to the finding of individual sheep that significantly exceed the chance level, we found a higher deviation in individual data towards performances with above 50% correct trials (more than 10 out of 20 trials correct) when indirect information was provided (‘empty’ condition) for goats than for sheep in experiment 2 (goats: eight out of 11 individuals or 73%; sheep: zero out of six individuals or 0%). Thus it is highly likely that, even with a higher sample size, goats would still outperform sheep at the group level.

When comparing specieś performances between experiments 1 and 2 only goats improved their performance when provided with indirect information, i.e., when only the content of the empty cup was shown to the subjects. This result shows that local enhancement at least affected the choice performance of goats [19]–[21]. Interestingly, the goats, especially in experiment 2, were more successful than the sheep in choosing the correct cup when the food was visible (‘both’ and ‘baited’ condition), indicating a better memorisation of where previously viewed food was hidden. However, further studies must be conducted to exclude other factors, e.g., different discriminatory abilities.

As mentioned in earlier studies of exclusion performance [20], [47], the test setup used in our experiments cannot distinguish between the different mechanisms that may have led to the increased performance by goats in the ‘empty’ condition across the two experiments. Since two goats in experiment 2 performed significantly above chance on an individual level in this condition, one may argue that these two subjects inferentially reasoned about the content of the baited cup solely by gathering information about the content of the empty cup [1]. However, without proper controls, a more parsimonious explanation for our findings is that the two individuals were simply avoiding the empty cup [20], [47]. Further studies should therefore implement modifications of the test procedure to control better for possible low-level explanations [15], [29], [48].

Referring to the *adaptive specialisation hypothesis* [33], different cognitive capabilities can be explained by species-specific adaptations to a specific feeding ecology [19], [20], [47]. According to the classification of ruminants after their feeding preferences [46], sheep are non-specialised high-fiber feeders (dietary grazers) and are less selective in their food intake. Goats, on the other hand, prefer low-fiber food (dietary browsers) and forage more selectively than sheep, e.g., feeding on a mixture of shrubs/herbs/forbs and grass and often switching seasonally [49]. We speculate that this higher flexibility may have led to the avoidance of a potential, but empty, food location in goats but not in sheep. In fact, an earlier study by Hosoi and colleagues [50] indicated avoidance of high-fiber food in goats but not in sheep when offered the option to feed on low-fiber food. In detail, goats responded to a losing situation (high fiber food) by increasing the frequency of shifting between two food patches. In contrast, sheep remained at the high fiber option, adopting a general win-stay/lose-shift strategy as it has been described for a number of mammalian species [51].

Additionally, other factors may have led to the differences in specieś decision-making. For instance, some goats, but not sheep, may have learned specific contingencies during the acquisition of the two experiments, as the goats had previous test experience with a visual discrimination task at the age of 6 months whereas the sheep did not. However, by comparing the first against the last ten trials of each condition, we only found an increase in the performance of goats when both cups were lifted and subjects therefore received full information of the content of the cups (‘both’ condition) in experiment 1. No other learning effects, especially in the crucial condition where subjects only received indirect information, i.e., information about the content of the empty cup, occurred. Importantly, the two goats that performed significantly above chance in this condition in experiment 2 did so from the very beginning of this experiment, by already choosing the correct cup in the first trial of that condition. Other important factors that could have influenced our results are differences in the specific ontogeny of test subjects [52]. As far as we know from their husbandry histories, the subjects of both species shared similar environments and were naïve with regard to object choice tasks. Both species were integrated in stable groups and were housed indoors on straw bedding. Because the testing times differed across species (goats: morning and afternoon; sheep: noon), one reasonable factor for the different performance may be a difference in motivation to participate in the task. Concentrated feed was provided to both groups in the early morning and early afternoon, so if anything, sheep, not goats, had a higher motivation to participate in both experiments, contrary to the finding obtained. Additionally, none of the sessions with goats or sheep had to be terminated due to an obvious decrease in motivation. In contrast, subjects remained highly motivated even for the last trials of two consecutive test sessions, as personal observations suggest.

Due to their high abundance in agriculture, goats and sheep appear to be promising candidate species for future studies on the influence of differing foraging strategies on cognitive capacities. Further research may investigate differences in risk sensitivity between these two species, as different feeding ecologies appear to have an impact on decision-making in other species, e.g., great apes [34], [35]. In conclusion, our results suggest that, at least on an individual level, goats rather than sheep, are able to solve a visible exclusion task and that this difference may reflect species-specific differences in species feeding ecology. In particular, flexibility in foraging behaviour may account for the different performances in the crucial ‘empty’ condition between goats and sheep. These differences in species feeding ecology should be taken into account as a potential explanatory factor when comparing exclusion performance across species.

## Supporting Information

Video S1 **Experiment 1 ‘empty’.** Trial of the condition ‘empty’ (Exp. 1) by subject ‘8’ (goat).
(MP4)

Video S2 **Experiment 2 ‘empty’.** Trial of the condition ‘empty’ (Exp. 2) by subject ‘8’ (goat).
(MP4)

Video S3 **Experiment 1 ‘both’.** Trial of the condition ‘both’ (Exp. 1) by subject ‘R2’ (sheep).
(WMV)
